# Radial cardiac T_2 _mapping with alternating T_2 _preparation intrinsically introduces motion correction

**DOI:** 10.1186/1532-429X-16-S1-P28

**Published:** 2014-01-16

**Authors:** Hélène Feliciano, Matthias Stuber, Ruud B van Heeswijk

**Affiliations:** 1 Radiology, University Hospital (CHUV), and University of Lausanne (UNIL), Lausanne, Switzerland; 2 Center for Biomedical Imaging (CIBM), Lausanne, Switzerland

## Background

**Figure 1:**
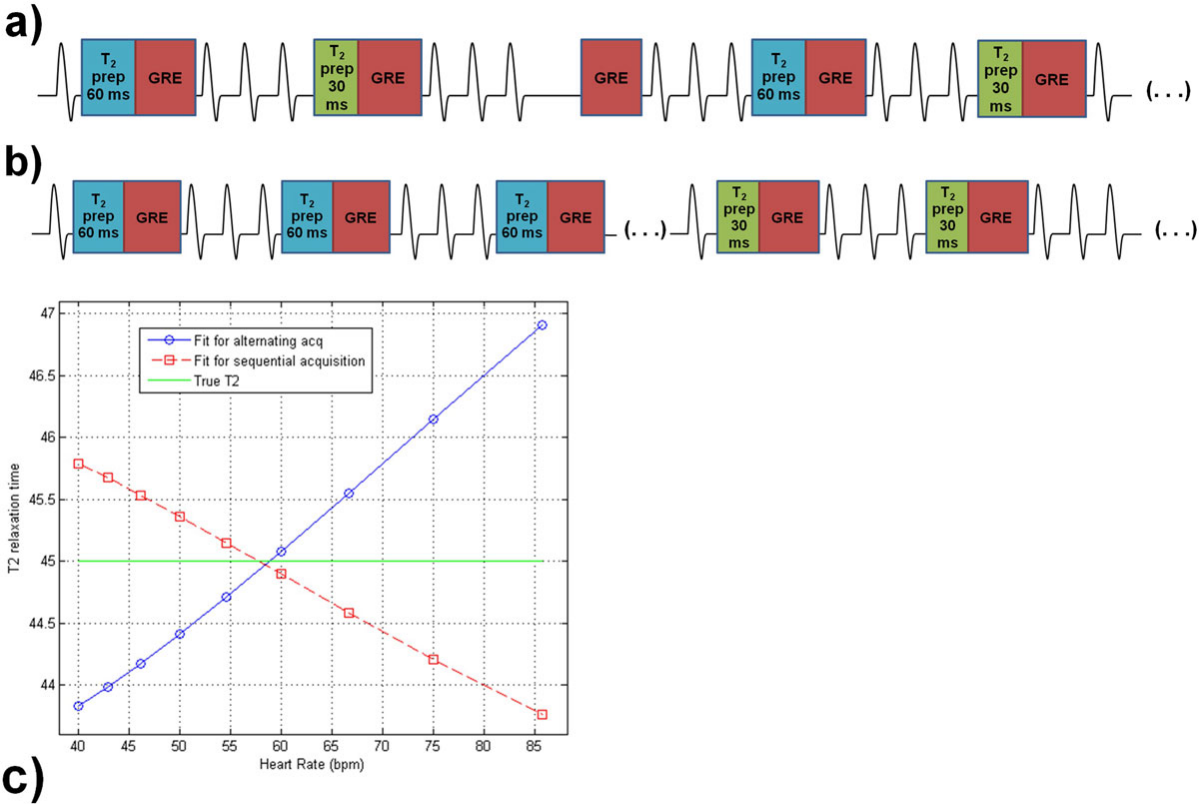
**a) Schematic of the alternating acquisition pattern**. The T_2_Prep duration is alternated between 60, 30 and 0 (no T_2_Prep) ms from heartbeat to heartbeat. All images are acquired in an interleaved fashion and on average experience similar motion. b) Schematic of the conventional sequential acquisition pattern. The T_2_Prep duration is changed only after acquisition of an image. This approach may be more vulnerable to irregular heart rates or respiration patterns. c) Simulations of stability of fitted T_2 _values for both methods against heart rate variation. For an input T_2 _of 45 ms, both the alternating acquisition (solid line) and the sequential acquisition (dashed line) result in a T_2 _variation of ~3 ms over the range of physiological heart rates.

T_2 _mapping through variation of the T_2 _preparation (T_2_Prep) duration has been increasingly used to robustly detect and quantify cardiac edema (Giri et al., JCardiovMagnReson2009). However, if images with incremental T_2_Prep duration are acquired in a sequential fashion, irregular breathing patterns and heart rates may adversely affect the quality of the T_2 _maps due to misalignment of the source images. A logical alternative is then to acquire all images in an alternating manner (Figure 1ab), where the T_2_Prep duration changes cyclically from one heartbeat to the next. Combined with a radial signal readout, this may minimize the vulnerability to respiratory or RR variability. We therefore simulated, implemented and tested the utility of an alternating magnetization preparation approach to T_2 _mapping.

## Methods

A navigator-gated ECG-triggered radial gradient-recalled-echo pulse sequence (20 lines per heartbeat, trigger every 3 heartbeats) was implemented to obtain source images for the T_2 _maps (van Heeswijk et al., JACCCardiovImag2012), with the possibility to apply the T_2_Prep durations of 60/30/0 ms in both an alternating and sequential manner. Bloch equation simulations were performed in order to estimate the fitting residual due to T_1 _relaxation (van Heeswijk et al., JACCCardiovImag2012) as well as the accuracy over a range of heart rates. The sequences were validated at 3T (12-channel surface coil array, on a Magnetom Trio, Siemens, Erlangen, Germany) in agar-NiCl_2 _phantoms by comparing the resulting T_2 _maps to gold-standard spin-echo (SE) T_2 _measurements. A mid-ventricular short-axis T_2 _map was then acquired with both pulse sequences in 7 healthy adult volunteers. The myocardial surface area was measured in the T_2 _maps, while a Student's t-test was applied to detect differences in T_2 _values and surface area.

## Results

**Figure 2:**
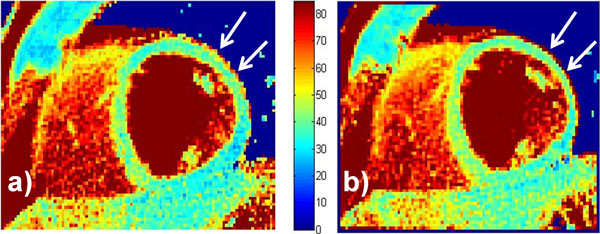
**T_2 _map of volunteer acquired with the alternating (a) and sequential (b) method**. Note that consistent with the quantitative findings, the antero-lateral myocardium is thicker when acquired with the alternating method (arrows).

The alternating sequence was as robust to heart rate variation as its sequential counterpart (Figure 1c), while its accuracy was confirmed in the phantoms (T_2 _= 45.4 ± 0.7 ms for the alternating method, vs 45.3 ± 0.7 ms for the sequential method and 45.1 ± 0.7 ms for the gold-standard). The myocardial surface area was increased in the alternated T_2 _maps of the volunteers (128 ± 24 cm^2 ^vs. 111 ± 20 cm^2^, p = 0.04) (Figure 2), while the average midventricular T_2 _value slightly differed between the alternated and sequential methods (T_2 _= 37.6 ± 6.6 ms alternated vs. 40.4 ± 6.1 ms sequential, p = 0.01).

## Conclusions

We successfully implemented and tested a T_2 _mapping methodology in which magnetization preparation is alternated. The *in vivo *T_2 _maps demonstrate that the alternated acquisition intrinsically aligns its sources images, resulting in a larger available myocardial surface, which in turn may allow for more accurate T_2 _value quantification.

## Funding

N/A.

